# Bis(2-methyl­pyridinium) tetra­chlorido­cuprate(II): synthesis, structure and Hirshfeld surface analysis

**DOI:** 10.1107/S2056989021006277

**Published:** 2021-06-25

**Authors:** Tahir Mehmood, Rajesh S. Bhosale, J. Prakasha Reddy

**Affiliations:** a Department of Chemistry, School of Sciences, Indrashil University, Rajpur, Gujarat, 382740, India

**Keywords:** 2-picoline complex, inorganic supra­molecular chemistry, crystal structure, hydrogen bonding

## Abstract

The title compound, (C_6_H_8_N)_2_[CuCl_4_], crystallizes in the monoclinic space group *I*2/*c*. The coordination around the copper atom is a distorted tetra­hedron. The 2-methyl­pyridinium ion (C_6_H_8_N^+^) inter­acts with the tetra­chloro­cuprate anion through N—H⋯Cl and C—H(phen­yl)⋯Cl contacts, forming a hydrogen-bonded layer-like structure. The supra­molecular structure is further stabilized by C—H(meth­yl)⋯Cl inter­actions between the layers.

## Abstract 2

The structure of bis­(2-methyl­pyridinium) tetra­chloro­cuprate(II) is characterized by layers formed by [C_6_H_8_N]^+^ and [CuCl_4_]^2–^ connected through N—H⋯Cl and C—H(phen­yl)⋯Cl inter­actions. The layers are further connected by C—H(meth­yl)⋯Cl inter­actions.

## Chemical context   

Supra­molecular organic and inorganic chemistry have been studied both from the fundamental as well as the application point of view, which is evident from the literature (Ziach *et al.*, 2018 ▸; Thorat *et al.*, 2013 ▸; Burslem *et al.*, 2016 ▸). With the surge in the number of compounds reported, potential applications of supra­molecular inorganic materials in energy storage, separation, catalysis, sensors, mol­ecular magnets, optoelectronic materials, *etc*., have attracted greater attention in recent years (Mueller *et al.*, 2006 ▸; Wan *et al.*, 2006 ▸; Férey *et al.*, 2003 ▸; James, 2003 ▸; Eddaoudi *et al.*, 2002 ▸; Ruben *et al.*, 2005 ▸, Kitagawa *et al.*, 2004 ▸, Stavila *et al.*, 2014 ▸). Because of the divergent combination of ligands and metal salts, an enormous number of structural architectures with different sizes and shapes could be constructed (Moulton & Zaworotko, 2001 ▸). The special characteristics and features such as ease of synthesis of the material, geometrically well-defined structures, exceptional tunability, post-synthetic modification, along with robustness of the material resulting from strong directional bonding, produce new opportunities and offer a unique platform amenable to the synthesis of more and more functional solids. For example, Adams *et al.* (2005 ▸) reported the synthesis of coordination compounds using a new synthetic route involving a thermal de­hydro­chlorination reaction in crystals of a pyridinium chloro­metallate bicomponent system, *i.e*., anionic metal complexes and organic cations.

As part of ongoing studies in our group (PrakashaReddy & Pedireddi, 2007 ▸; Reddy *et al.*, 2014 ▸), the synthesis of coord­ination complexes using pyridine ligands has been reported. Hence, we further extended our studies to utilize the pyrid­in­ium ligand and to study *in situ* the single-crystal-to-single-crystal transition (SCSCT) to investigate the reaction mechanism. In our endeavours to synthesize new functional solids, using a transition-metal anion and a pyridinium cation, we have chosen the CuCl_2_ and 2-methyl­pyridinium salt complex. Herein, we report the synthesis and crystal structure of a bis­(2-methyl­pyridinium) tetra­chloro­cuprate coordination complex.
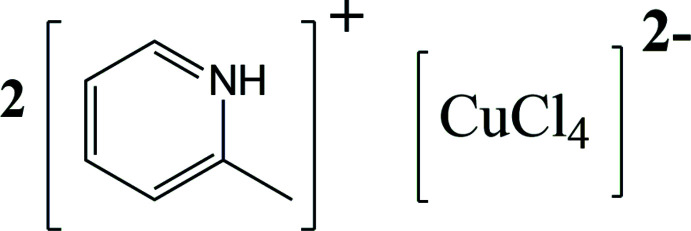


## Structural commentary   

**Figure 1:**
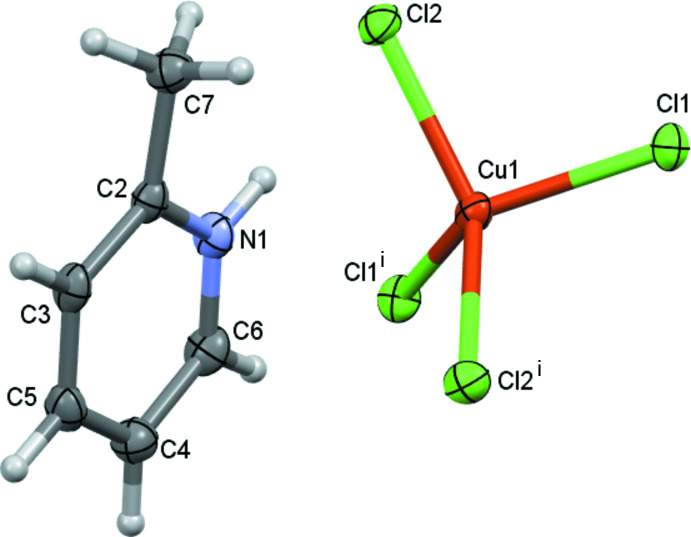
The mol­ecular structure of the title compound, showing the atom labelling and displacement ellipsoids drawn at the 50% probability level. Hydrogen atoms are shown as small spheres of arbitrary size. [Symmetry code: (i) 1 − *x*, *y*, 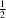 − *z*.]

The title complex crystallizes in the monoclinic space group *I*2/*c*. Since the Cu^2+^ cation occupies a special position, the asymmetric unit consists of a 2-methyl­pyridinium cation, [2-Me(Py)H]^+^, and half of a tetra­chloro­cuprate(II) anion, [CuCl_4_]^2–^. The mol­ecular structure of the complex along with the atom-labelling scheme is shown in Fig. 1 ▸. Each copper center is four-coordinated by chlorine anions and adopts a distorted tetra­hedral geometry. Structural analysis shows that the Cl—Cu—Cl angles vary from 98.55 (2) to 137.4 (3)° with four angles smaller and two larger than the standard tetra­hedral angle. A plausible reason for a larger deviation from the standard 109.5° might be due to the N—H⋯Cl and C—H⋯Cl inter­actions. A similar marked deviation from the standard tetra­hedral angle has been previously observed by other research groups (Wyrzykowski *et al.*, 2011 ▸; Jasrotia *et al.*, 2018 ▸). The Cu—Cl bond lengths [Cu1—Cl1 = 2.250 (1) Å, Cu1—Cl2 = 2.249 (1) Å] agree well with those reported for other structures (Marsh *et al.*, 1982 ▸; Dodds *et al.*, 2018 ▸; Molano *et al.*, 2020 ▸; Reddy, 2020 ▸). The intra­molecular C_ar_—C_ar_ bond lengths in the [2-Me(Py)H]^+^ fall in the range 1.370 (3)–1.395 (3) Å. The N1—C2 and N1—C6 bond lengths are 1.350 (2) and 1.346 (2) Å, respectively.

## Supra­molecular features and Hirshfeld surface analysis   

**Figure 2:**
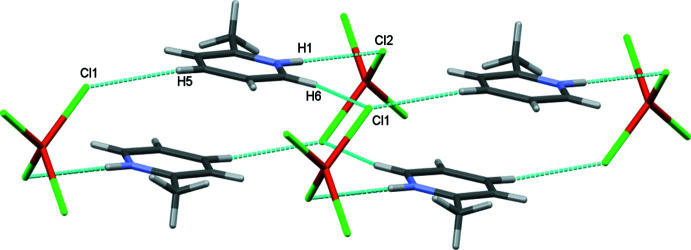
The N—H⋯Cl and C—H⋯Cl inter­actions between cations and anions in the crystal structure of the title compound.

**Figure 3:**
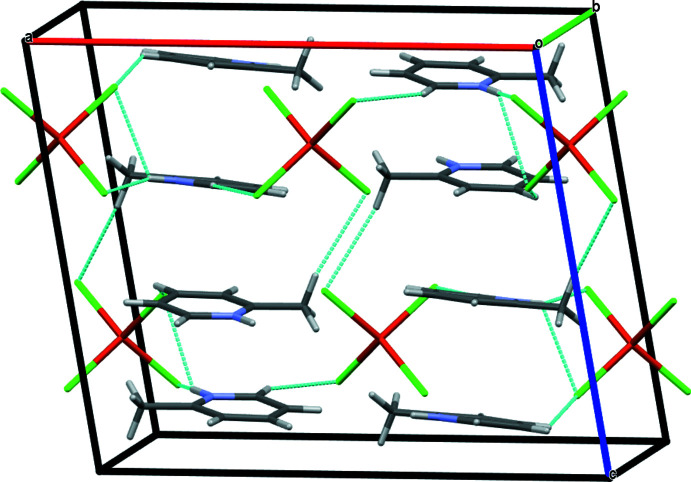
The crystal packing of the title compound viewed along the *b* axis with inter­molecular contacts shown as dashed lines.

In the crystal, complex mol­ecules related by the twofold rotation axis are connected by pairs of N—H⋯Cl and C_ar_—H⋯Cl inter­actions through a protonated N and an aromatic hydrogen attached to the carbon atom with the chloride ligand bonded to copper, forming a monomeric unit. These units inter­act with adjacent ones through C_ar_—H⋯Cl hydrogen bonding (Table 1 ▸, Fig. 2 ▸). The N—H⋯Cl and C—H⋯Cl distances and associated bond angles lie within the ranges observed for other similar inter­actions reported in the literature (Adams *et al.*, 2005 ▸; Vittaya *et al.*, 2015 ▸; Wyrzykowski *et al.*, 2011 ▸; Jasrotia *et al.*, 2018 ▸). The supra­molecular structure is further stabilized by C_meth­yl_—H⋯Cl inter­actions involving hydrogens of the methyl group and chlorides bonded to copper, generating layers along the crystallographic *b* axis (Fig. 3 ▸).

**Figure 4:**
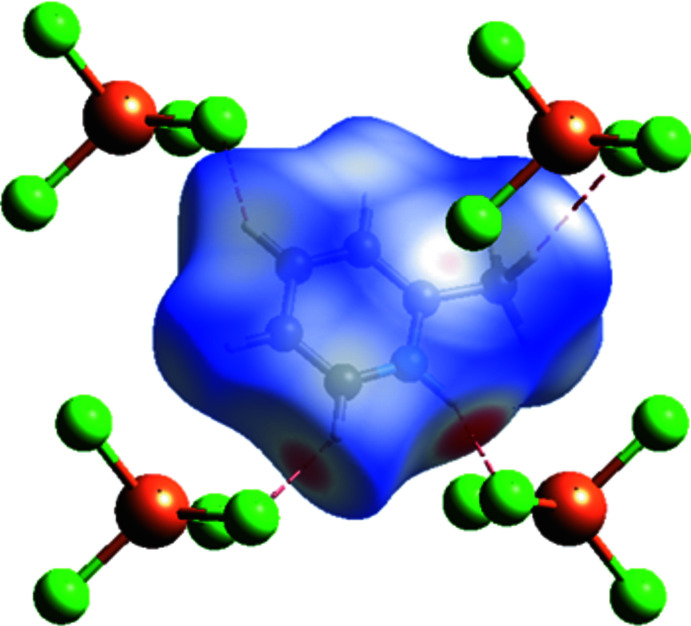
Hirshfeld surface mapped over *d*_norm_ highlighting the regions of N—H⋯Cl and C—H⋯Cl inter­molecular contacts.

**Figure 5:**
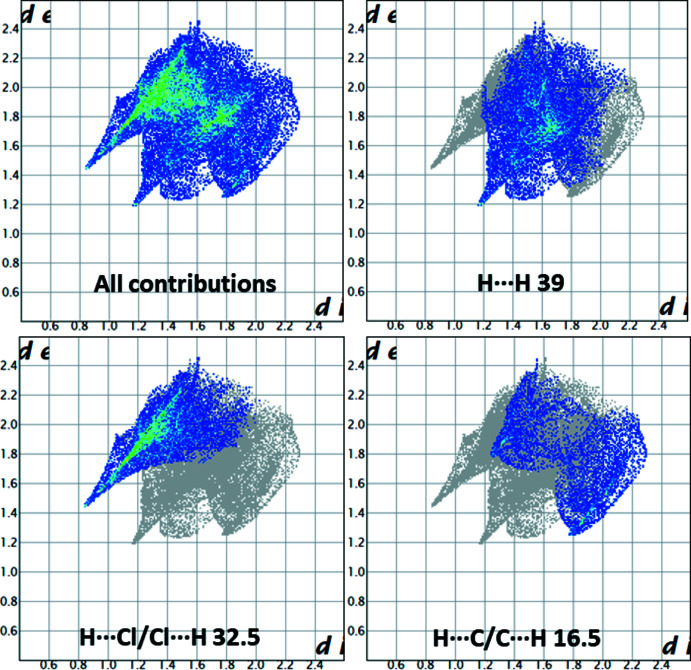
The full two-dimensional fingerprint plot for the organic cation in the title compound and those delineated into H⋯H (39%), Cl⋯H/H⋯Cl (32.5%) and C⋯H/H⋯C (16.5%) contacts.

To further investigate the inter­molecular inter­actions present in the title compound, a Hirshfeld surface analysis was performed and the two-dimensional fingerprint plots were generated with *Crystal Explorer17* (Turner *et al.*, 2017 ▸). The Hirshfeld surface mapped over *d*_norm_ and corresponding colours representing various inter­actions are shown in Fig. 4 ▸. The red points on the surface correspond to the N—H⋯Cl and C—H⋯Cl inter­actions. The two-dimensional fingerprint plots (McKinnon *et al.*, 2007 ▸) are shown in Fig. 5 ▸. On the Hirshfeld surface, the largest contribution (53.1%) comes from the weak van der Waals H⋯H contacts. The inter­action of *d*_norm_ on the two-dimensional fingerprint plot shows two spikes; each one corresponds to H⋯H (39%) and H⋯Cl/Cl⋯H (32.5%) respectively. The H⋯Cl inter­action highlights the hydrogen bond between adjacent moieties in the crystal structure. The C⋯H/H⋯C (16.5%) inter­actions appear as two shoulders. These inter­actions play a crucial role in the overall stabilization of the crystal packing.

## Database survey   

A search of the Cambridge Structural Database (CSD, Version 5.41, update of August 2020; Groom *et al.*, 2016 ▸) revealed two related complexes containing 2-methyl­pyridinium: [2-methyl­pyridinium tetra­chloro­ferrate(III)] (CCDC refcode WAYJEN; Wyrzykowski *et al.*, 2011 ▸) and [bis­(2-methyl-pyridinium) tetra­chloro-zinc(II)] (CCDC refcode WIPCUW; Jasrotia *et al.*, 2018 ▸). The mol­ecular structures of both WAYJEN and WIPCUW display three-dimensional supra­molecular networks arising from N—H⋯Cl and C—H⋯Cl inter­actions. In addition, the search also revealed a 2-methyl­pyridine and copper chloride complex: [di­chloro-bis­(2-methyl­pyridine)Cu(II)] (CCDC refcode CMPYCU01; Marsh *et al.*, 1982 ▸) and [aqua-di­chloro-bis­(2-methyl­pyridine)Cu(II)] (CCDC refcode BIJWUM; Marsh *et al.*, 1982 ▸) and a very recently published di­chlorido­methano­lbis(2-methyl­pyridine)Cu(II) complex (Reddy, 2020 ▸). All of these structures display three-dimensional supra­molecular networks stabilized by C—H⋯Cl and O—H⋯Cl inter­actions.

## Synthesis and crystallization   

Both 2-methyl­pyridine and anhydrous copper(II) chloride obtained from Aldrich were used for the reaction. Anhydrous copper(II) chloride (0.495 g, 0.005 mol) was dissolved in 10 ml of distilled water. To this solution, 2-methyl­pyridine (0.93 g, 0.01 mol) was added followed by addition of few drops of HCl (36%) and the resulting mixture was stirred for ∼30 min. at room temperature. The solution was then allowed to stand at room temperature for a few hours before being filtered and left at room temperature for crystallization. Block-shaped, pale-yellow-coloured crystals were obtained after 36 h.

## Refinement   

Crystal data, data collection and structure refinement details are summarized in Table 2 ▸. H atoms were placed in calculated positions with C—H = 0.93–0.96 Å and N—H = 0.88 Å and refined as riding with fixed isotropic displacement parameters [*U*_iso_(H) = 1.2–1.5*U*_eq_(C, N)].

## Supplementary Material

Crystal structure: contains datablock(s) I. DOI: 10.1107/S2056989021006277/dj2020sup1.cif

Structure factors: contains datablock(s) I. DOI: 10.1107/S2056989021006277/dj2020Isup2.hkl

CCDC reference: 2090586

Additional supporting information:  crystallographic information; 3D view; checkCIF report

## Figures and Tables

**Table 1:** Hydrogen-bond geometry (Å, °)

*D*—H⋯*A*	*D*—H	H⋯*A*	*D*⋯*A*	*D*—H⋯*A*
N1—H1⋯Cl1^i^	0.88	2.93	3.4297 (16)	118
N1—H1⋯Cl2^i^	0.88	2.41	3.2050 (16)	150
C6—H6⋯Cl1^ii^	0.95	2.62	3.453 (2)	147
C7—H7*A*⋯Cl2	0.98	2.92	3.850 (2)	159
C7—H7*B*⋯Cl2^iii^	0.98	2.98	3.872 (2)	151

Symmetry codes: (i) -x+1, -y, -z+1; (ii) x+{\script{1\over 2}}, y-{\script{1\over 2}}, z+{\script{1\over 2}}; (iii) -x+1, -y+1, -z+1.

**Table 2:** Experimental details

Crystal data	
Chemical formula	(C_6_H_8_N)_2_[CuCl_4_]
*M*_r_	393.62
Crystal system, space group	Monoclinic, *I*2/*c*
Temperature (K)	120
*a*, *b*, *c* (Å)	15.2354 (8), 8.3683 (3), 12.8372 (6)
β (°)	99.205 (5)
*V* (Å^3^)	1615.60 (13)
*Z*	4
Radiation type	Mo *K*α
μ (mm^−1^)	2.00
Crystal size (mm)	0.32 × 0.27 × 0.25

Data collection	
Diffractometer	Agilent Xcalibur, Sapphire3
Absorption correction	Analytical (*CrysAlis PRO*; Agilent, 2014 ▸)
*T*_min_, *T*_max_	0.848, 0.965
No. of measured, independent and observed [*I* > 2σ(*I*)] reflections	24556, 2821, 2324
*R*_int_	0.071
(sin θ/λ)_max_ (Å^−1^)	0.758

Refinement	
*R*[*F*^2^ > 2σ(*F* ^2^)], *wR*(*F* ^2^), *S*	0.036, 0.081, 1.08
No. of reflections	2821
No. of parameters	88
H-atom treatment	H-atom parameters constrained
Δρ_max_, Δρ_min_ (e Å^−3^)	0.58, −0.58

Computer programs: *CrysAlis PRO* (Agilent, 2014 ▸), *SUPERFLIP* (Palatinus & Chapuis, 2007 ▸; Palatinus & van der Lee, 2008 ▸; Palatinus *et al.*, 2012 ▸), *SHELXL* (Sheldrick, 2015 ▸) and *OLEX2* (Dolomanov *et al.*, 2009 ▸).

## supplementary crystallographic information

### Crystal data

**Table:**

(C_6_H_8_N)_2_[CuCl_4_]	*F*(000) = 796
*M**_r_* = 393.62	*D*_x_ = 1.618 Mg m^−^^3^
Monoclinic, *I*2/*c*	Mo *K*α radiation, λ = 0.71073 Å
*a* = 15.2354 (8) Å	Cell parameters from 5462 reflections
*b* = 8.3683 (3) Å	θ = 2.9–32.5°
*c* = 12.8372 (6) Å	µ = 2.00 mm^−^^1^
β = 99.205 (5)°	*T* = 120 K
*V* = 1615.60 (13) Å^3^	Blocks, pale yellow
*Z* = 4	0.32 × 0.27 × 0.25 mm

### Data collection

**Table:**

Agilent Xcalibur, Sapphire3 diffractometer	2324 reflections with *I* > 2σ(*I*)
Detector resolution: 16.1511 pixels mm^-1^	*R*_int_ = 0.071
ω scans	θ_max_ = 32.6°, θ_min_ = 2.9°
Absorption correction: analytical (CrysAlisPro; Agilent, 2014)	*h* = −22→22
*T*_min_ = 0.848, *T*_max_ = 0.965	*k* = −11→12
24556 measured reflections	*l* = −19→19
2821 independent reflections	

### Refinement

**Table:**

Refinement on *F*^2^	Primary atom site location: iterative
Least-squares matrix: full	Hydrogen site location: inferred from neighbouring sites
*R*[*F*^2^ > 2σ(*F*^2^)] = 0.036	H-atom parameters constrained
*wR*(*F*^2^) = 0.081	*w* = 1/[σ^2^(*F*_o_^2^) + (0.0308*P*)^2^ + 1.4147*P*] where *P* = (*F*_o_^2^ + 2*F*_c_^2^)/3
*S* = 1.08	(Δ/σ)_max_ < 0.001
2821 reflections	Δρ_max_ = 0.58 e Å^−^^3^
88 parameters	Δρ_min_ = −0.58 e Å^−^^3^
0 restraints	

### Special details

**Table:**

Geometry. All esds (except the esd in the dihedral angle between two l.s. planes) are estimated using the full covariance matrix. The cell esds are taken into account individually in the estimation of esds in distances, angles and torsion angles; correlations between esds in cell parameters are only used when they are defined by crystal symmetry. An approximate (isotropic) treatment of cell esds is used for estimating esds involving l.s. planes.

### Fractional atomic coordinates and isotropic or equivalent isotropic displacement parameters (Å^2^)

**Table:**

	*x*	*y*	*z*	*U*_iso_*/*U*_eq_	
Cu1	0.500000	0.22261 (4)	0.250000	0.01825 (9)	
Cl1	0.38953 (3)	0.10590 (5)	0.13863 (3)	0.02196 (11)	
Cl2	0.41898 (3)	0.32033 (5)	0.36739 (4)	0.02260 (11)	
N1	0.68501 (11)	0.01294 (19)	0.63521 (12)	0.0218 (3)	
H1	0.642734	−0.054296	0.645210	0.026*	
C2	0.66434 (12)	0.1697 (2)	0.62543 (13)	0.0197 (3)	
C3	0.73176 (13)	0.2747 (2)	0.61076 (14)	0.0222 (4)	
H3	0.720458	0.386273	0.605979	0.027*	
C4	0.83354 (14)	0.0533 (2)	0.61276 (16)	0.0261 (4)	
H4	0.890816	0.012622	0.607122	0.031*	
C5	0.81548 (14)	0.2165 (2)	0.60311 (15)	0.0247 (4)	
H5	0.861102	0.288380	0.591178	0.030*	
C6	0.76632 (14)	−0.0468 (2)	0.63060 (15)	0.0254 (4)	
H6	0.777088	−0.158092	0.639704	0.031*	
C7	0.57039 (13)	0.2174 (2)	0.62851 (16)	0.0259 (4)	
H7A	0.536395	0.214343	0.556960	0.039*	
H7B	0.569141	0.325976	0.656845	0.039*	
H7C	0.543936	0.143189	0.673767	0.039*	

### Atomic displacement parameters (Å^2^)

**Table:**

	*U*^11^	*U*^22^	*U*^33^	*U*^12^	*U*^13^	*U*^23^
Cu1	0.02142 (16)	0.01465 (15)	0.01929 (16)	0.000	0.00510 (12)	0.000
Cl1	0.0253 (2)	0.0174 (2)	0.0226 (2)	−0.00165 (16)	0.00223 (17)	−0.00185 (15)
Cl2	0.0276 (2)	0.0172 (2)	0.0246 (2)	0.00035 (16)	0.00935 (17)	−0.00340 (16)
N1	0.0268 (8)	0.0154 (7)	0.0235 (7)	−0.0023 (6)	0.0054 (6)	0.0011 (6)
C2	0.0264 (9)	0.0172 (8)	0.0155 (8)	−0.0004 (7)	0.0033 (7)	0.0000 (6)
C3	0.0311 (10)	0.0150 (8)	0.0203 (8)	−0.0030 (7)	0.0040 (7)	0.0004 (6)
C4	0.0251 (10)	0.0266 (10)	0.0267 (9)	0.0016 (8)	0.0040 (8)	−0.0010 (8)
C5	0.0281 (10)	0.0221 (9)	0.0244 (9)	−0.0069 (7)	0.0056 (8)	−0.0008 (7)
C6	0.0326 (10)	0.0172 (9)	0.0265 (10)	0.0031 (7)	0.0048 (8)	0.0016 (7)
C7	0.0263 (10)	0.0243 (10)	0.0276 (10)	0.0018 (7)	0.0061 (8)	0.0004 (8)

### Geometric parameters (Å, º)

**Table:**

Cu1—Cl1^i^	2.2496 (5)	C3—C5	1.383 (3)
Cu1—Cl1	2.2496 (5)	C4—H4	0.9500
Cu1—Cl2	2.2492 (4)	C4—C5	1.395 (3)
Cu1—Cl2^i^	2.2492 (4)	C4—C6	1.370 (3)
N1—H1	0.8800	C5—H5	0.9500
N1—C2	1.350 (2)	C6—H6	0.9500
N1—C6	1.346 (2)	C7—H7A	0.9800
C2—C3	1.387 (3)	C7—H7B	0.9800
C2—C7	1.493 (3)	C7—H7C	0.9800
C3—H3	0.9500		
			
Cl1—Cu1—Cl1^i^	128.54 (3)	C5—C4—H4	121.0
Cl2^i^—Cu1—Cl1	99.614 (17)	C6—C4—H4	121.0
Cl2^i^—Cu1—Cl1^i^	98.550 (17)	C6—C4—C5	118.10 (19)
Cl2—Cu1—Cl1	98.549 (17)	C3—C5—C4	120.61 (18)
Cl2—Cu1—Cl1^i^	99.616 (17)	C3—C5—H5	119.7
Cl2^i^—Cu1—Cl2	137.36 (3)	C4—C5—H5	119.7
C2—N1—H1	118.0	N1—C6—C4	119.91 (18)
C6—N1—H1	118.0	N1—C6—H6	120.0
C6—N1—C2	124.00 (17)	C4—C6—H6	120.0
N1—C2—C3	117.46 (17)	C2—C7—H7A	109.5
N1—C2—C7	117.88 (17)	C2—C7—H7B	109.5
C3—C2—C7	124.64 (17)	C2—C7—H7C	109.5
C2—C3—H3	120.1	H7A—C7—H7B	109.5
C5—C3—C2	119.88 (17)	H7A—C7—H7C	109.5
C5—C3—H3	120.1	H7B—C7—H7C	109.5
			
N1—C2—C3—C5	2.2 (3)	C6—N1—C2—C3	−0.6 (3)
C2—N1—C6—C4	−1.5 (3)	C6—N1—C2—C7	177.92 (17)
C2—C3—C5—C4	−1.7 (3)	C6—C4—C5—C3	−0.4 (3)
C5—C4—C6—N1	2.0 (3)	C7—C2—C3—C5	−176.27 (18)

Symmetry code: (i) −*x*+1, *y*, −*z*+1/2.

### Hydrogen-bond geometry (Å, º)

**Table:**

*D*—H···*A*	*D*—H	H···*A*	*D*···*A*	*D*—H···*A*
N1—H1···Cl1^ii^	0.88	2.93	3.4297 (16)	118
N1—H1···Cl2^ii^	0.88	2.41	3.2050 (16)	150
C6—H6···Cl1^iii^	0.95	2.62	3.453 (2)	147
C7—H7*A*···Cl2	0.98	2.92	3.850 (2)	159
C7—H7*B*···Cl2^iv^	0.98	2.98	3.872 (2)	151

Symmetry codes: (ii) −*x*+1, −*y*, −*z*+1; (iii) *x*+1/2, *y*−1/2, *z*+1/2; (iv) −*x*+1, −*y*+1, −*z*+1.
